# Processing Impact on Performance of Solid Dispersions

**DOI:** 10.3390/pharmaceutics10030142

**Published:** 2018-08-30

**Authors:** Dan Zhang, Yung-Chi Lee, Zaher Shabani, Celeste Frankenfeld Lamm, Wei Zhu, Yongjun Li, Allen Templeton

**Affiliations:** 1 Pharmaceutical Sciences, Merck Research Laboratories, Merck & Co., Inc., Kenilworth, NJ 07033, USA; wei_zhu3@merck.com (W.Z.); yongjun.li@merck.com (Y.L.); allen_templeton@merck.com (A.T.); 2 Regulatory Affairs, Merck Research Laboratories, Merck & Co., Inc., Kenilworth, NJ 07033, USA; zaher.shabani@merck.com (Z.S.); celeste.frankenfeld@merck.com (C.F.L.)

**Keywords:** solid dispersion, bioavailability, spray drying, hot melt extrusion, poorly water-soluble compound, in vitro and in vivo characterization

## Abstract

The development of a weakly basic compound is often challenging due to changes in pH that the drug experiences throughout the gastrointestinal tract. As the drug transitions from the low pH of the stomach to the higher pH of the small intestine, drug solubility decreases. A stomach with a higher pH, caused by food or achlorhydric conditions brought about by certain medications, decreases even the initial solubility. This decreased drug solubility is reflected in lower in vivo exposures. In many cases, a solubility-enabling approach is needed to counteract the effect of gastrointestinal pH changes. Solid dispersions of amorphous drug in a polymer matrix have been demonstrated to be an effective tool to enhance bioavailability, with the potential to mitigate the food and achlorhydric effects frequently observed with conventional formulations. Because solid dispersions are in a metastable state, they are particularly sensitive to processing routes that may control particle attributes, stability, drug release profile, and bioperformance. A better understanding of the impacts of processing routes on the solid dispersion properties will not only enhance our ability to control the product properties, but also lower development risks. In this study, a weakly basic compound with greatly reduced solubility in higher pHs was incorporated into a solid dispersion via both spray drying and hot melt extrusion. The properties of the solid dispersion via these two processing routes were compared, and the impact on dissolution behavior and in vivo performance of the dispersions was investigated.

## 1. Introduction

Enhancing bioavailability remains a key challenge to oral formulation development because of increasing numbers of poorly water-soluble compounds that are brought forward for development. After a drug is administered orally, it disintegrates, dissolves, and then permeates through the membranes of the gastrointestinal (GI) tract to reach systemic circulation. Therefore, to make an insoluble compound orally bioavailable, a critical step is to achieve a fast dissolution and to maintain a high concentration of drug in the gastrointestinal milieu [1,2]. Over the years, various approaches have been developed to enhance the bioavailability of poorly water-soluble drugs, such as salt formation [3,4], nanoformulations [5], liquid capsule formulations [6,7], and amorphous formulations (neat drug or drug dispersed in polymer matrix) [8,9]. These approaches take advantage of solubility enhancement, particle size reduction, wettability improvement, or a combination of some of these features. In recent years, amorphous solid dispersion has been widely used as an effective formulation approach for enhancing the bioavailability of poorly solubilizing drugs due to its adaptability to a wide range of compounds [10]. Its value has been realized in helping deliver poorly water-soluble compounds, such as Belsomra, Zepatier, Orkambi, Novir, and other commercial products [11,12].

Since the beginning of the research of amorphous formulations more than 50 years ago, various hypotheses have been proposed regarding the mechanism of bioavailability improvement through solid dispersions, stemming from the Noyes-Whitney equation [13]. First, in the amorphous state, the energy barrier is lower for a molecule to cross from the solid state to the solution state. This usually results in an enhanced apparent solubility and potentially supersaturation and fast dissolution. Second, in a solid dispersion, the drug and the polymer can form a molecular assembly, reducing the drug particle size to increase the dissolution rate. Third, the polymer can improve the wettability of the compound surface to facilitate dissolution. Fourth, the polymer can inhibit crystallization of the amorphous drug to a state with lower solubility and slower dissolution. Furthermore, these actions of solid dispersions can potentially mitigate the achlorhydric effect, one of the root causes of low bioavailability observed from basic compounds [14,15].

Several different approaches have been used to make amorphous solid dispersions, including hot melt extrusion, spray drying, spray congealing, and co-precipitation [16,17,18,19,20,21]. The two most commonly used approaches in the pharmaceutical industry are hot melt extrusion and spray drying. A typical spray-drying process involves atomization of a solution containing an active pharmaceutical ingredient (API) and polymer into a spray of droplets of micron sizes. This is followed by rapid evaporation of solvent from the droplets upon contact with heated processing gas (typically air or nitrogen) in a drying chamber. The formation of dry particles proceeds under controlled temperature and airflow conditions [22,23]. Hot melt extrusion is another robust and continuous process to produce molecular dispersions of drugs into a polymer matrix. During this process, a powder or granule mixture of polymeric components and active substances, including any additives or plasticizers, are blended, melted, and extruded through an orifice or die under controlled temperature, pressure, feed rate, and screw speed [24]. The resultant extrudate is milled to an appropriate particle size for further formulation processing.

Compared with hot melt extrusion (HME), spray-dried dispersions (SDD) have some unique advantages, including a relatively short development time and versatility for a wide range of polymers. In the early stage of development, SDDs can be manufactured using a sub-gram quantity of drug substance via a bench top spray-drier, and can be scaled up to kilogram or metric ton quantities in later-stage development, establishing a line of sight from preclinical to clinical to commercialization stages. Besides its amenability to thermally labile compounds, antioxidant can be easily incorporated into SDDs to minimize oxidation when combined with the use of an inert nitrogen drying gas. The disadvantages of SDDs include variability in granule attributes (particle size, bulk/tapped density, and flowability), which depend on the scale of the spray drier and changes in downstream manufacturing processes throughout the development of a product. Furthermore, the spray-drying solvent must be carefully selected as it may have an impact on throughput, process conditions, stability, and potentially the homogeneity of SDD granules due to solubility differences between the drug and polymer in the solvent.

In contrast to spray drying, hot melt extrusion can produce high-density and flowable granules that lead to simpler downstream manufacturing processes with lower potential for change throughout the manufacturing scales. Although it is difficult to apply HME in early development, it is particularly well-suited for large-scale manufacturing. Compared to spray drying, hot melt extrusion does not require the use of organic solvents; thus, it is a “green” process with a smaller plant footprint, making it more cost-effective with fewer safety/environmental concerns. However, the granules from HME usually have low compactibility, which presents challenges to tablet formulations. In addition, it is not a viable process for thermally labile compounds or polymers.

Because solid dispersions are in a metastable state and thus are a state function of the processing, they are strongly impacted by the manufacturing process. In particular, the processing route may control properties of solid dispersions, such as particle attributes, stability, drug release profile, and bioperformance. A better understanding of impacts of processing routes on the solid dispersion properties will not only enhance our ability to control the product properties, but also lower development risk from the discovery phase to the last stage of development and commercialization. However, there are limited data available.

An exploratory model compound MK-A, was under development for the treatment of cancer, which is a therapeutic area that attracts intensive research in the pharmaceutical industry; for example, some recently approved cancer drugs are vemurafenib, ribociclib, and ivosidenib. MK-A exists in a non-hygroscopic crystalline free base form with a single melting endotherm at 133 °C. Although the free base form is physically and chemically stable, it is poorly soluble in water as well as gastrointestinal media. To enhance the aqueous solubility of MK-A, the free base was converted to a crystalline HCl salt form. However, much of the HCl salt that is solubilized at low pH precipitates as free base when it transitions to a higher pH (such as in the transition from stomach to small intestine). Key properties of MK-A are summarized in Table 1 below.

Therefore, the aim of this study is to use MK-A as a model compound to compare the performance of solid dispersions prepared via spray drying or hot melt extrusion. A systematic approach was adapted for the overall formulation development. The initial solid dispersion was prepared via the spray-drying process. Solutions with different types of polymers and different drug loads were sprayed. Biorelevant dissolution was used to rank the performance of the spray-dried solid dispersion intermediates. Based on the rank order of biorelevant dissolution results, selected compositions were further interrogated via both spray drying and hot melt extrusion. Additional physical and chemical stability and in vivo animal studies were conducted to understand the impact of manufacturing process on the overall performance of solid dispersions.

## 2. Materials and Methods

### 2.1. Materials

The HCl salt and free base of MK-A were manufactured by Merck & Co. Inc. (Kenilworth, NJ, USA). HPLC-grade acetone (Sigma-Aldrich, St. Louis, MO, USA), HPMCAS (Hypromellose Acetate Succinate) MF and HF grade (Shin-Etsu Chemical Co., Totowa, NJ, USA), and Kollidon VA 64 (vinylpyrrolidone-vinyl acetate copolymer) (BASF, Florham Park, NJ, USA) were purchased. The microcrystalline cellulose (Avicel PH 102), spray dried lactose (Fast-Flo 316), silicon dioxide (Cab-o-sil), and Swedish Orange size 00 hard gelatin capsule shell were obtained from FMC (Philadelphia, PA, USA), Foremost Farms (Baraboo, WI, USA), Cabot (Boston, MA, USA), and Capsugel (Greenwood, SC, USA), respectively. All materials were used as received.

### 2.2. Methods

#### 2.2.1. Spray Drying Dispersions Preparation

The polymer (HPMCAS-M, HPMCAS-H, or VA-64) and API (ranging from 3:1 to 3:2 of polymer to API ratios) were dissolved in acetone at 5% solid loading. The mixture was stirred on a magnetic stir plate or submersed into a Branson Ultra-Sonicator until all components were dissolved. A ProCepT Micro-Spray Dryer with a bifluid nozzle of orifice of 0.6 mm, (9060 Zelzate, Belgium) was used in this study [25]. Nitrogen was used as the drying gas. The atomization pressure (L/min), feed rate (mL/min), drying gas flow rate (m^3^/min), inlet temperature (°C), and outlet temperature (°C) were adjusted to 6, 6, 0.35, 85, and 53, respectively. The harvested spray dried materials were further oven-dried (40 °C under reduced pressure) overnight to remove residual solvent.

#### 2.2.2. Hot Melt Extrusion Preparation

A 16 mm twin-screw extruder (Thermo Fisher Scientific, Grand Island, NY, USA) with the all-conveying screw configuration (5 forwarding 30 and 5 forwarding 60) was utilized for the extrusion. A pre-blended mixture consisting of drug to HPMCAS (M or H) at a 1:3 ratio was fed into the extruder via a K-Tron twin-screw feeder. Ten grams per minute of blend was fed into the extruder with 250 rpm and the barrel temperature was controlled between 130 °C and 150 °C depending on the grade of the polymer used. The extrudate was air-cooled and milled via a Fitzmill for downstream evaluation.

#### 2.2.3. Formulation Preparation for PK Studies

The HME capsules (150 mg potency) were prepared by combining the HME intermediate with crospovidone (disintegrant) at a 10:1 ratio. The resulting blend was hand-filled into hard gelatin capsules.

The spray-dried dispersion capsules (75 mg potency) were prepared by combining the spray-dried intermediate with crospovidone (disintegrant) at a 10:1 ratio. The resulting blend was hand-filled into hard gelatin capsules.

The spray-drying suspension formulation was made by suspending the spray-dried dispersion powder in pH 4.0 citrate buffer at a drug concentration of 10 mg/mL.

The HCl salt solution formulation was prepared by dissolving MK-A HCl salt in a 10% tween 80 solution to reach a drug concentration of 4.3 mg/mL, with a resultant pH of 4.4.

The dry-filled capsule (DFC) formulation of the HCl salt was prepared by blending the drug with lactose monohydrate (diluent), microcrystalline cellulose (binder), crospovidone (disintegrant), colloidal silicon dioxide (glidant), and stearic acid (lubricant). The resulting blend was roller-compacted and milled into granules, which were then filled into hard gelatin capsules.

#### 2.2.4. In Vitro Dissolution

Dissolution was conducted using bathless dissolution instruments (Distek Premiere 5100, North Brunswick, NJ, USA) with apparatus 2 (paddles) operated at 100 rpm. Dissolution medium was warmed to and maintained at 37 °C ± 0.5 °C. Dissolution was performed upon either free solid dispersion granules or granules and blends encapsulated and placed within a sinker. For single-step dissolution, 500 mL of medium was used. For two-stage dissolution, the first stage was conducted in 250 mL, and then 250 mL of the second medium was added for a total of 500 mL. Approximately 2 mL were sampled at each timepoint and then immediately syringe-filtered (1 μm glass filter, Acrodisc) or ultracentrifuged at 80,000 rpm for 15 min on a Beckman Coulter Optima Max-XP Ultracentrifuge.

Dissolution media were prepared as follows. All chemicals were purchased through Fisher Scientific (Fair Lawn, NJ, USA) unless otherwise noted.

- FaSSIF (fasted state simulated intestinal fluid) was prepared from Phares SIF powder (Croyden, Surrey, UK), following their recommended composition, dissolved in a buffer of sodium dihydrogen phosphate, sodium chloride, and sodium hydroxide, adjusted to pH 6.5.
- SGF (simulated gastric fluid) was prepared from a preparation of 0.034 M sodium chloride adjusted to pH 1.8 with concentrated hydrochloric acid [26].
- For two-stage dissolution, a 2× FaSSIF medium was prepared at twice the concentration of standard FaSSIF [27].
- 1 µm filtration and 80 K spin down were used for the sample preparation.

#### 2.2.5. In Vivo Dog PK Study

Fasted male beagle dogs were used to study the bioperformance of MK-A formulations. Studies were conducted under a protocol approved by the Institutional Animal Care and Use Committee (IACUC) of Merck & Co. Inc., West Point, PA, USA (IACUC Number: 10078544080244; start date: 1 July 2010). Six male beagle dogs with body weight between 7 and 11 kg were assigned to two different dosing groups and administered a test formulation according to the scheme shown Table 2.

Animals were fasted overnight and food was given after the 4-h blood collection. Each animal received 0.05 mL of the pentagastrin solution (0.12 mg/mL) per kg of body weight as an intramuscular injection approximately 30 min before dosing. SDD and HME capsules were dosed orally followed by a tap water rinse administered via oral gavage at a volume of 3.5 mL/kg. The solution formulation was dosed by oral gavage at a volume of 3.5 mL/kg. The SDD suspension was dosed by oral gavage at 15 mL per dog. After dosing, the beaker was rinsed with 10 mL of water twice and both rinses were dosed through the gavage tube for a total volume of 35 mL. Water was removed before dosing, and was returned 1 h after dosing to mimic dosing in the clinic. Blood was collected at pre-dose, 15 min, 30 min, 1, 2, 4, 6, 8, and 24 h post-dose. No adverse effect was observed for the study animals during or post-dose.

MK-A concentration in dog plasma samples were analyzed by LC/MS/MS with lower limit of quantification (LLOQ) of 20 ng/mL. Area under the curve (AUC _0–24 h_), observed maximum plasma concentration (*C*_max_), and time of *C*_max_ (*T*_max_) were calculated using a linear trapezoidal, non-compartmental model of WinNonLin v5.2. Means and standard deviation were calculated using WinNonLin v5.2. Plasma profiles were generated using WinNonLin v5.2 or Microsoft^®^ Excel 2003. Concentrations below LLOQ were set at zero for calculation purposes.

## 3. Results and Discussion

### 3.1. The Need for an Enabled Formulation

**Figure 1:**
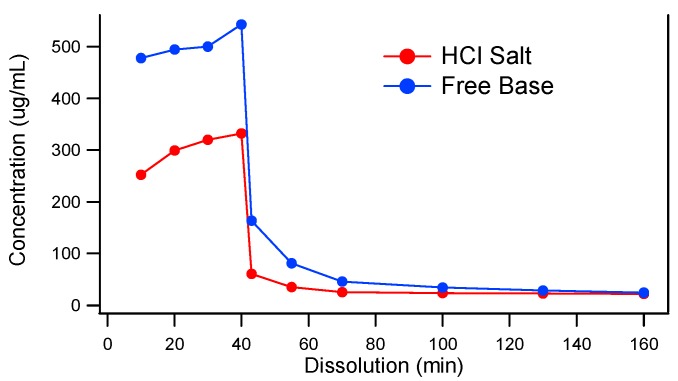
Two-stage dissolution of MK-A HCl Salt and Free Base.

Because the model compound, MK-A, is a weak base, it is important to understand the precipitation tendency of MK-A upon pH increase. Two-stage dissolution testing (mimicking gastric conditions for 30 to 45 min followed by a change in pH to mimic the small intestine environment for at least 2 h) can be used as a tool to project the potential in vivo performance of oral solid dosage forms [27]. The two-stage dissolution profiles of MK-A free base and its HCl salt are displayed in Figure 1. As can be seen from the figure, under the acidic condition (0–40 min), the free base and HCl salt of MK-A have a higher concentration of 543 and 333 µg/mL, respectively. Upon the pH change at 40 min, the drug precipitates and the concentrations of both forms of API are reduced to 24 µg/mL. These results suggest that MK-A has a strong precipitation tendency upon pH increase, and the initial dissolution (drug dissolved) achieved in gastric fluid may not strongly enhance the bioperformance. It should also be noted that the lower drug concentration of the HCl salt in the stomach pH could be due to the common ion effect.

The data from the two-stage dissolution clearly indicated that an alternative delivery approach should be explored to reduce the precipitation tendency of the drug upon pH change in the GI tract. It was proposed that by dispersing the API into a crystallization inhibitor as a solid dispersion might help achieve supersaturation to improve in vivo exposure. Spray drying and hot melt extrusion, two commercially proven solid dispersion production platforms, were evaluated. VA-64 and HPMCAS, two commonly used polymers, were chosen for solid dispersion studies.

**Figure 2:**
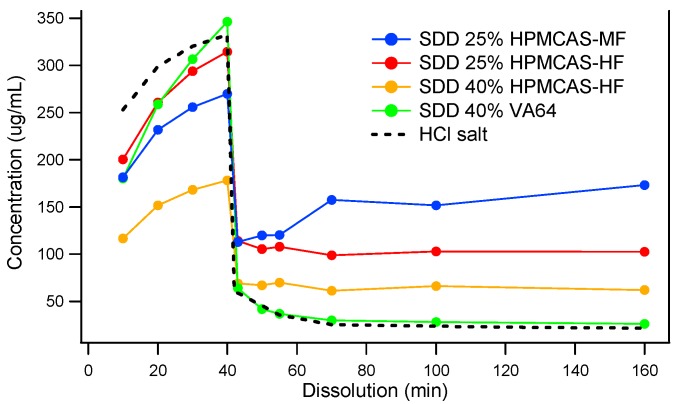
Two-stage dissolution of MK-A solid dispersions prepared by spray drying.

Since MK-A was in the preclinical exploratory stage and a limited amount of drug substance was available, spray drying was used to screen the type and grade of polymers. Spray-dried dispersions (SDDs) were manufactured using VA64 and two grades of HPMCAS (MF and HF) at 25% and 40% drug loads. Figure 2 shows the dissolution profiles of four distinct solid dispersions: 25% MK-A/HPMCAS-HF, 40% MK-A/HPMCAS-HF, 25% MK-A/HPMCAS-MF, and 40% MK-A/VA64. Although 40% MK-A/VA64 SDD achieves the highest concentration in SGF, it shows little supersaturation in FaSSIF. This is an expected result due to the non-enteric and hydrophilic nature of VA64. Interestingly, under the acidic condition, MK-A should not have released from HPMCAS-based formulations due to their enteric nature. However, MK-A did release from the HMPCAS-based solid dispersions regardless of the grade of HPMCAS and the level of drug load. This could be due to the small particle size of the spray-dried material without the formation of the enteric coating layer. This will be discussed in more detail in later sections. Nevertheless, the use of HPMCAS did provide some supersaturation after the pH transition from 1.2 to 6.5. The 25% MK-A/HPMCAS-MF dispersion provided the greatest solubility enhancement after being transferred from SGF to FaSSIF medium (approximately a 7-fold increase compared to the pure API HCl salt).

Based on the dissolution data from Figure 2, the 25% drug load HPMCAS-MF and -HF were selected for feasibility evaluation in hot melt extrusion. A total of four solid dispersion compositions were evaluated based on physical and chemical stability, sufficient solubilization capacity to mitigate a potential pH effect on solubility reduction upon transition from gastric to small intestine pH conditions, and enhancing exposure in the preclinical animal model.

### 3.2. Comparison of In Vitro Dissolution of Spray-Dried and HME Solid Dispersions

**Figure 3:**
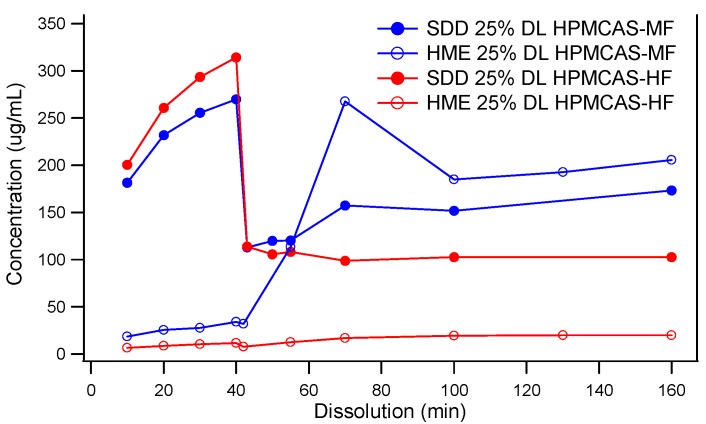
Two-Stage dissolution of MK-A solid dispersions prepared by spray drying or hot melt extrusion (Powders of the solid dispersions were used for the dissolution. For HME dispersions, the original HME extrudates were milled to a particle size of 213 μm). DL, drug load.

Two-stage dissolution profiles of HPMCAS-MF-based spray-dried and hot melt extrusion solid dispersions are shown in Figure 3. Although the components and drug load were the same across both, vastly different dissolution behaviors were observed. Unlike the SDD, the HME released little to no drug in the SGF stage. The lack of release at low pH did not diminish the solubility enhancement in FaSSIF (pH 6.5) in HME made with HPMCAS-MF. However, HME based on HPMCAS-HF never released a significant portion of drug in the FaSSIF stage. It is known that the dissolution of HPMCAS depends on the ratio of acetate to succinate groups, and the ratio is different between HPMCAS-MF and -HF. However, at pH 6.5, the HPMCAS-HF material was expected to dissolve and release the drug. It is possible that this unusual behavior was due to the slow kinetics of dissolution of the large particle size of the HME extrudates, as discussed below, but it is not fully understood at this point. In contrast to the HME dispersions, the dissolution of the SDDs was markedly different in the SGF stage, as both SDDs based on the MF and HF grades of HPMCAS released a significant portion of the API. In addition, both spray-dried dispersions provided significant solubility enhancement in the FaSSIF stage relative to the crystalline API. However, the HPMCAS-MF-based SDD displayed superior in vitro performance.

### 3.3. The Impact of Solid Dispersion Properties on In Vitro Dissolution

**Figure 4:**
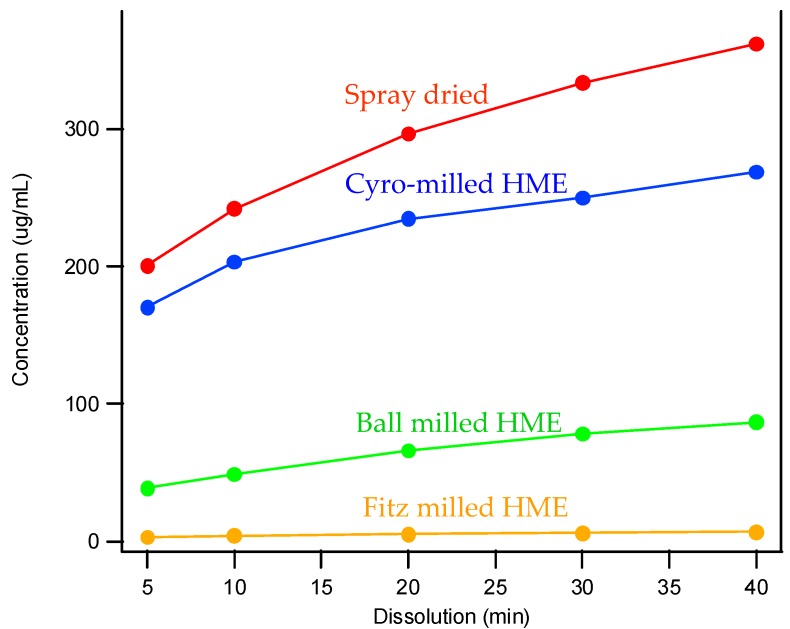
Dissolution profiles of SDD and milled HMEs in simulated gastric fluid (SGF).

When considering the marked difference in dissolution profiles of the same composition (25% drug load (DL) HPMCAS-MF spray-dried dispersion versus 25% DL HPMCAS-MF HME dispersion), it is important to note the different properties of the spray-dried granules versus the hot melt granules, such as density, particle size, and surface area. These properties can drastically impact the dissolution profile. As shown in Table 3, the HME particles were significantly larger than the SDD granules, resulting in a much smaller surface-area-to-drug-content ratio, and the different drug release profiles of the two dispersions could be attributed to this difference. To gain a better understanding of the impact of particle size on dissolution, the HME particles were milled to different sizes using different milling techniques, and the dissolution profile in SGF was compared to that of the SDD (Figure 4). The rank order of dissolution matched the particle size of each dispersion (spray-dried particles being the smallest, and Fitz-milled HME the largest), indicating that surface area plays an important role in dissolution of the solid dispersions. Since the cryo-milled HME and the spray-dried dispersion have particle size in the same range, comparable dissolution profiles were obtained in the SGF stage for both solid dispersions.

### 3.4. Stability of Solid Dispersions

#### 3.4.1. Chemical Stability

The stability of various formulations of MK-A was evaluated under accelerated conditions (Table 4). Levels of potency reduction right after the solid dispersion preparation were 1.7% and 4.6% for spray drying and hot melt extrusion, respectively. The higher potency reduction in HME was attributed to the higher processing temperature of the HME process (~140 °C) at which thermal degradation of the drug occurred. The potency of the amorphous form of active was further reduced after 2 weeks storage at 40 °C/75% relative humidity (RH) conditions in closed glass vials by up to as much as 8.5% loss of potency in the solid dispersion via HME. Data also suggest that the initial potency reduction from the spray-drying process and subsequent potency loss on stability can be mitigated by using nitrogen as the drying gas as well as adding an antioxidant.

#### 3.4.2. Physical Stability

The physical stability of solid dispersion samples was evaluated by powder X-ray diffraction (PXRD) and modulated differential scanning calorimetry (mDSC). No sign of recrystallization was observed for up to 40% drug load in the HPMCAS-based solid dispersion. Unlike the HPMCAS dispersions, the VA64 composition demonstrated signs of phase separation based on the DSC data. This suggests that VA64 might not be a suitable polymer for MK-A solid dispersion preparation. The HPMCAS-based solid dispersions were physically stable for at least 2 weeks under 40 °C and 75% relative humidity storage conditions. In addition, data showed that the addition of antioxidant did not impact the physical stability of the HPMCAS-based composition.

### 3.5. In Vivo Study

**Figure 5:**
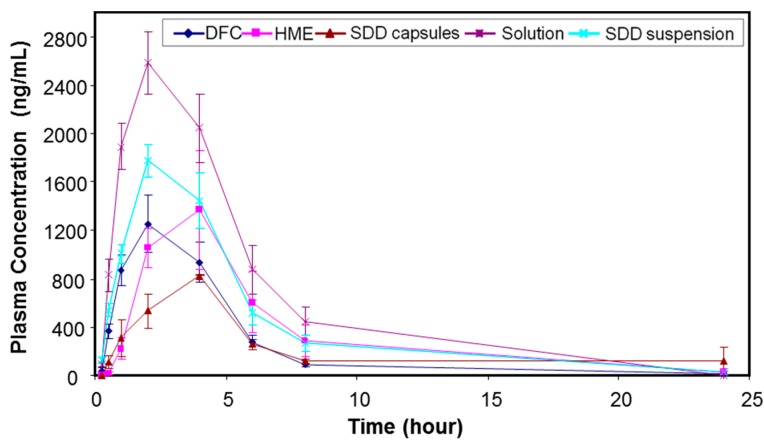
Profiles of mean (±SE) plasma concentration versus time after administration of MK-A formulations (15 mpk) to pentagastrin-pretreated male Beagle dogs (*n* = 3 or 6).

All the in vitro performance results indicated that the spray-drying process with 25% drug load HPMCAS-MF would be a viable option to achieve a higher relative solubility and maintain acceptable chemical and physical stability. On the other hand, the hot melt extrusion process generated higher impurity levels, but also reached higher supersaturation. Thus, both spray-dried and hot melt extrusion HPMCAS-MF-based formulations with 25% drug load were tested in a dog model to assess their in vivo performance. For comparison, a solution (MK-A HCl salt in 10% tween 80), a spray-dried suspension, and a simple dry-blend of HCl salt with commonly used excipients, such as microcrystalline cellulose, lactose, and crospovidone (DFC), were also dosed into the dog model as control formulations. Table 5 summarizes the mean pharmacokinetic parameters after administration of the above formulations to pentagastrin-pretreated male Beagle dogs at 15 mg/kg dose (*n* = 3 or 6). The purpose of pentagastrin pretreatment is to lower the dogs’ gastric pH to ~2 for 1 h to simulate human gastric pH [28]. Profiles of mean plasma concentration versus time are shown in Figure 5.

Using the mean PK data in pentagastrin-pretreated dogs, the HME capsules, SDD capsules, SDD suspension, and solution formulation dosed at 15 mpk provided an exposure that was 1.33-fold, 0.81-fold, 1.58-fold, and 2.40-fold of the DFC, respectively. Mean AUC_0–24 h_ were estimated at 12.4 µM·h, 7.6 µM·h, 14.7 µM·h, and 22.4 µM·h, respectively. The *C*_max_ of HME capsules, SDD capsules, SDD suspension, and solution formulation was 1.22-fold, 0.65-fold, 1.44-fold, and 2.05-fold of the DFC, respectively. The median *T*_max_ was 2 h, 4 h, 2 h, 2 h, and 2 h, respectively, for the five tested formulations. The solution formulation provided an exposure which was 2.4-fold of the DFC, suggesting a prolonged supersaturation of API during gastric-intestinal transition, which enhanced the absorption of the drug.

**Figure 6:**
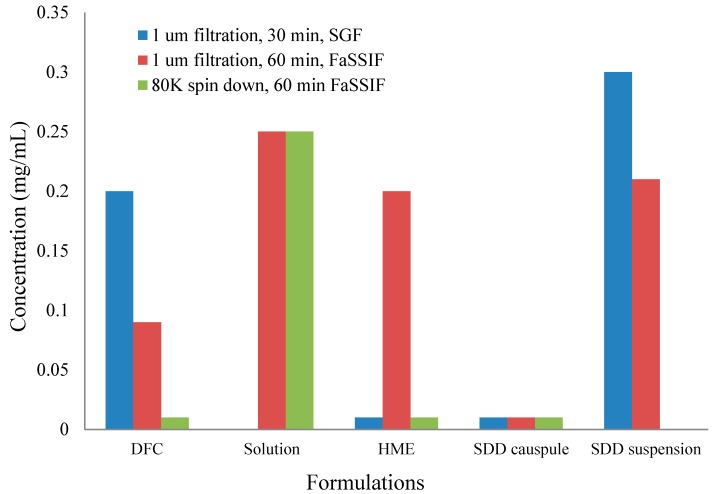
1-μm filtration and 80 K ultracentrifugation results of two-stage dissolution of different formulations. FaSSIF, fasted state simulated intestinal fluid.

To understand the in vivo data of SDD and HME formulations, two-stage dissolution was performed on the same formulations as the in vivo studies. The results are shown below in Figure 6. In order to detect nanoparticle formation during dissolution [29], samples were prepared with 1 μm filtration and 80 K centrifugation. Filtration removed any large, undissolved particles but allowed nanoparticles to remain in the filtrate for analysis. Further centrifugation at 80,000 rpm spun down nanoparticles, leaving only pure solution. After analysis of both samples (filtrate and pure solution), the faction of drug in pure solution versus the amount present in nanoparticles was calculated. From Figure 6, the solution formulation shows very similar dissolution profiles independent on the clearance methods. This confirms that there were no nanoparticles formed in the solution formulation. On the other hand, the HME in capsules demonstrates significant difference between filtered and ultracentrifuged samples, indicating nanoparticles were formed. The SDD in capsules did not release a significant amount of drug with either a 1-μm filter or 80 K ultracentrifugation as the clearance method. This is due to the compaction of the spray-dried granules within the capsule, which created a solid plug. Due to the lack of disintegration of the SDD in the capsule, dissolution of a suspension of the SDD was compared. As shown in Figure 6, similar to HME, the SDD suspension also demonstrates formation of nanoparticles. Because a significant difference between filtered and ultracentrifuged samples was observed in the HME and SDD formulations, it is clear that the solubility enhancement observed with the solid dispersions compared to the crystalline drug is due to nanoparticle formation, and not simply greater solubility of the amorphous drug.

Significantly different profiles were observed using 80 K ultracentrifugation and 1-µm filtration for HME capsules, indicating that the modest increase of in vivo exposure observed for the HME formulation (33% increase against DFC) was mainly due to the formation of nanoparticles. The SDD capsules, however, showed a slight decrease of in vivo exposure (19% decrease against DFC). The in vitro dissolution of SDD capsules showed minimal formation of nanoparticles for up to 120 min. Observation of dissolution vessel showed that a solid core was formed and remained almost intact, which prevented the release of HPMCAS even in FaSSIF and the formation of nanoparticles.

The SDD suspension in dogs delineated the disintegration issue of the SDD capsule formulation, as we observed a moderate increase of exposure against DFC (58%) at a 15 mpk dose. The exposure of the SDD suspension was also slightly higher than that of the HME capsules and appeared to have faster onset. This higher exposure is likely due to the smaller particle size of SDD that leads to earlier solubilization of API in the stomach as observed in the two-stage dissolution study. However, the exposure of all solid dispersion formulations was lower than that of the solution formulation.

In summary, the exposure of HME capsules and SDD suspension was 1.33-fold and 1.58-fold of that of DFC following dosing in fasted pentagastrin-pretreated dogs, respectively, indicating a moderate increase of exposure for solid dispersion formulations at a 15 mpk dose.

## 4. Conclusions

In this study, two commonly used solid dispersion approaches, spray drying and hot melt extrusion, were utilized to produce solid dispersions of the model compound MK-A. HPMCAS-MF was selected as the stabilizing polymer based on its physical/chemical stability and in vitro performance. The main finding of this study is that the final processing of the enabled API and the resulting physical characteristics have a profound impact upon the in vitro and in vivo performance. For MK-A, the MF grade of the stabilizing polymer HPMCAS provided the most supersaturation and solubility enhancement in both spray-dried and hot melt extrusion solid dispersions. In addition, a moderate drug load of 25% outperformed the higher drug load of 40%. The SDD clearly shows the advantage of API chemical stability compared to the HME dispersion. In in vitro experiments, HME and SDD demonstrate very different drug release profiles, particularly in the SGF stage. This is because solid dispersion properties, such as density, particle size, and surface area, have a profound impact on in vitro dissolution. Although showing a marked difference in in vitro dissolution profiles, HME and SDD show similar in vivo performance and a moderate increase of exposure. The in vivo performance enhancement is likely due to nanoparticle formation during dissolution of the solid dispersions.

## Figures and Tables

**Table 1:** Physical/chemical properties of MK-A.

**Chemical Structure**	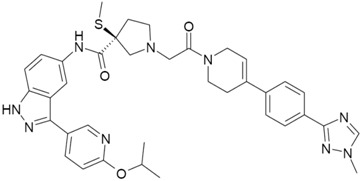	
**Drug Form**	HCl Salt Anhydrous Crystalline Form	Free Base Hydrate Crystalline Form
**Melting Point (** **°** **C)**	249	133
**pKa**	5.9 and 2.2	
**Log P (free base)**	4.5	
**BCS Classification**	II	
**Solubility (µg/mL)**	H_2_O: 661 (pH = 3.5) Simulated Gastric Fluid (SGF): 525 Fasted Simulated Small Intestine Fluid (FaSSIF): 21	H_2_O: 0.3 (pH = 7.3) Simulated Gastric Fluid (SGF): 907 Fasted Simulated Small Intestine Fluid (FaSSIF): 2
**Stability**	Chemically stable in the solid-state; Physically stable at various temperatures and humidity conditions but sensitive to mechanical stress	Chemically and physically stable

**Table 2:** Dosing Scheme for the dog study of MK-A.

Period	Group A (*n* = 3)	Group B (*n* = 3)
1	HME capsules	SDD capsules
2	Solution	HME capsules
3	SDD suspension	SDD suspension

HME, hot melt extrusion; SDD, spray-dried dispersion.

**Table 3:** Morphology and particle size of 25% DL HPMCAS-MF solid dispersions at 150× magnification.

Solid Dispersions	Morphology (SEM)	Particle Size (μm)
Spray dried	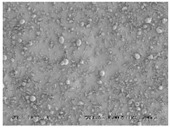	7
Fitz-milled HME	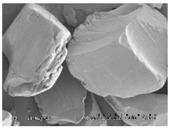	213
Ball-milled HME	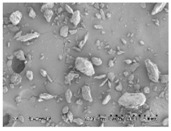	59
Cryo-milled HME	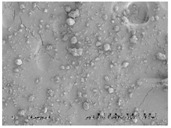	16

**Table 4:** Physical/Chemical Stability of MK-A Amorphous Solid Dispersions prepared by Spray Drying or Hot Melt Extrusion.

Polymer	Condition	Initial (% Area)	2 Weeks at 40 °C/75% RH Closed (% Area)	2 Weeks at 40 °C/75% RH Closed Physical Form
MK-A (FB)	-	98.8	98.8	-
SDD HPMCAS-HF	25% DL	96.7	90.3	Amorphous
SDD HPMCAS-HF	40% DL	96.8	89.8	Amorphous
SDD HPMCAS-MF	25% DL	97.1	91.8	Amorphous
SDD VA64	40% DL	96.9	89.3	Amorphous/Phase separation
SDD HPMCAS-LF	25% DL under N_2_	98.8	95.4	Amorphous
* SDD HPMCAS-LF	25% DL under N_2_ antioxidants	98.9	98.2	Amorphous
HME HPMCAS-HF	25% DL, 140 °C	95.1	86.6	Amorphous
HME HPMCAS-MF	25% DL, 136 °C	94.2	86.0	Amorphous

* Antioxidants: BHT (0.1%)/BHA (0.1%).

**Table 5:** Average pharmacokinetic parameters (mean ± standard error (SE)) after administration of MK-A formulations (15 mpk) to pentagastrin-pretreated male Beagle dogs (*n* = 3 or 6).

Formulation	Dose (mpk)	AUC_0–24 h_ (µM × h)	*C*_max_ (µM)	*T*_max_ (h)	AUC Ratio (Versus DFC)
HME capsules (*n* = 6)	15	12.4 ± 2.65	2.22 ± 0.42	2.0 (2.0–4.0) *	1.33
SDD capsules (*n* = 3)	15	7.59 ± 1.44	1.19 ± 0.00	4.0 (4.0–4.0) *	0.81
SDD suspension (*n* = 6)	15	14.7 ± 2.13	2.62 ± 0.19	2.0 (2.0–4.0) *	1.58
Solution (*n* = 3)	15	22.4 ± 3.41	3.73 ± 0.38	2.0 (2.0–2.0) *	2.40
DFC (Benchmark, *n* = 6)	15	9.35 ± 1.34	1.82 ± 0.34	2.0 (1.0–2.0) *	1.00

* For *T*_max_, the median value is provided along with the range in parenthesis; AUC: area under curve; DFC: dry-filled capsule.
